# Stump Appendicitis After Appendectomy: A Case Report

**DOI:** 10.7759/cureus.90740

**Published:** 2025-08-22

**Authors:** Luis F Ochoa, Carlos A Garay, Alexis J Lira, Mauro A Cardenas, Miguel Rodriguez

**Affiliations:** 1 Surgery, Hospital General Instituto de Seguridad y Servicios Sociales de los Trabajadores del Estado (ISSSTE) Presidente General Lázaro Cárdenas del Río, Chihuahua, MEX; 2 Surgery, Hospital General de Tijuana, Tijuana, MEX; 3 Surgery, Instituto Mexicano del Seguro Social Unidad Médica de Alta Especialidad (UMAE) 71, Torreon, MEX; 4 Surgery, Universidad de Oriente, Houston, USA

**Keywords:** abdominal pain, appendectomy, appendix, appendix stump, stump

## Abstract

Acute appendicitis is one of the most common causes of emergency surgery worldwide. Its diagnosis is primarily based on clinical presentation, supported by laboratory tests such as a complete blood count and imaging studies, with a non-contrast CT scan considered the gold standard. Although appendectomy is generally safe, it carries the risk of immediate and delayed complications. One such complication, stump appendicitis, is rare and often underrecognized, which can delay diagnosis and treatment. The objective of this report is to highlight the importance of considering stump appendicitis in patients with a prior history of appendectomy who present with symptoms suggestive of acute appendicitis. Prompt recognition is crucial to prevent severe complications, including peritonitis secondary to intestinal perforation, which can increase morbidity and mortality. We present the case of a 48-year-old male from Chihuahua, Mexico, who arrived at our hospital’s emergency department with a four-day history of abdominal pain localized predominantly in the right iliac fossa. His medical history included an appendectomy performed several years earlier, which initially led clinicians to consider alternative diagnoses.

## Introduction

Appendicitis is an inflammation of the vermiform appendix, typically caused by obstruction of the appendiceal lumen due to fecaliths, lymphoid hyperplasia, infection, or, less commonly, neoplasms. It remains the leading cause of emergency abdominal surgery worldwide, with peak incidence occurring in the second and third decades of life [1].

Stump appendicitis is a rare complication characterized by acute inflammation of residual appendiceal tissue left behind after an appendectomy. It occurs when a portion of the appendix, the “stump”, is inadvertently retained during the initial surgery, later becoming obstructed and inflamed. This condition mimics the clinical presentation of primary appendicitis, despite the patient’s history of prior appendectomy. Its estimated incidence is approximately 1 in 50,000 cases of appendectomy [2-4].

Patients commonly present with right lower quadrant abdominal pain, leukocytosis, and imaging findings similar to those seen in primary appendicitis. Diagnosis is frequently delayed due to low clinical suspicion in patients with a prior appendectomy [5,6]. The main risk factor is leaving a long appendiceal stump, usually longer than 0.5-0.65 mm; also, failure to invaginate the stump, complicated surgery or difficult dissection of the appendix are factors to take into account [7]. 

It is a rare and underestimated entity, especially when classic clinical symptoms are present but there is a history of appendectomy; it leads to initial differential diagnoses, delaying treatment and leading to fatal complications. Here, we present the case of an adult male with right lower quadrant pain, fever, and a history of appendectomy. Stump appendicitis was diagnosed via computed tomography (CT), and the patient underwent emergency exploratory laparotomy.

## Case presentation

This is a 48-year-old male who attends the emergency department with a four-day history of continuous abdominal pain localized to the right lower quadrant. The pain was accompanied by two episodes of diarrhea, and the patient also reported having a fever on two occasions, with temperatures exceeding 38°C. The patient stated that he received treatment from a general practitioner with butyl hyoscine and metoclopramide, which did not improve his condition. Upon admission, a physical examination was performed with the following findings: scar from a previous appendectomy, absence of bowel sounds, abdominal distension with positive rebound tenderness, positive McBurney's sign, positive Psoas sign, positive Obturator sign, as well as signs of peritoneal irritation (involuntary rigidity of the abdomen, pain to fine, superficial touch).

Laboratory and imaging tests

**Figure 1:**
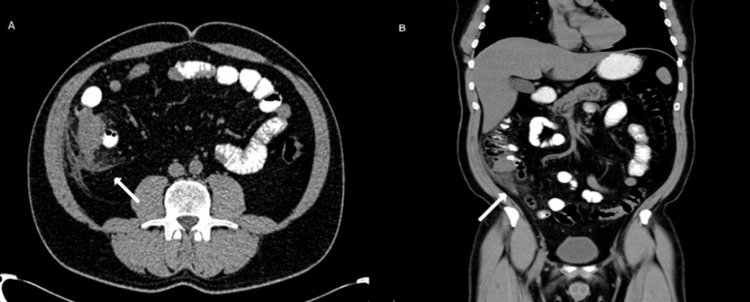
Abdominal CT scan Figure A: Axial view of abdominal CT scan: Inflammatory changes can be observed around the pericecal fat with thickening of the area. Figure B: Coronal view of abdominal CT scan: Loss of pericolic fat definition at the level of the cecum is evident (arrows).

Laboratory results showed signs of systemic inflammatory response: leukocytosis of 14.55 cells/mm³ (reference range 4.5-10.5) without neutrophilia 8.93 cells/mm³. Due to the clinical presentation, an oral contrast-enhanced abdominal CT scan was performed, which revealed changes in the pericolic fat in the cecal region, as well as the presence of a phlegmon in such area (Figures 1A, 1B).

Differential diagnosis

The attending surgeon aimed to rule out constipation, gastroenteritis, and/or right-sided diverticulitis, all of which were ruled out through the oral contrast-enhanced CT scan. The history of a previous appendectomy, the duration of symptoms, the pain in the lower right quadrant accompanied by intestinal symptoms, signs of an acute abdomen, and markers of systemic inflammatory response open up a range of possibilities. 

Treatment

**Figure 2:**
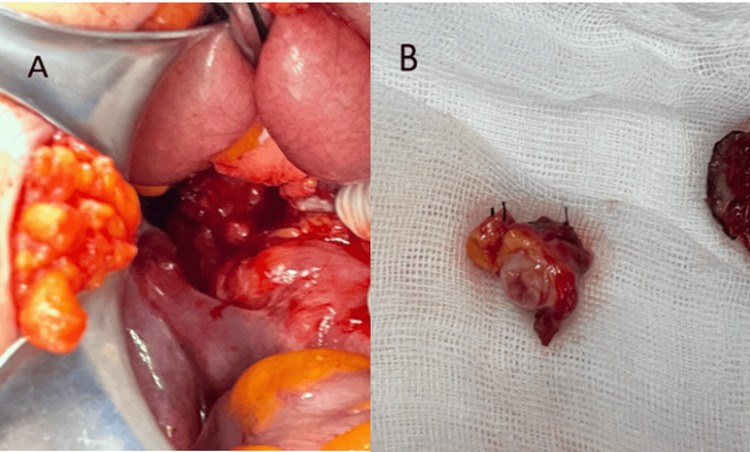
Macroscopic findings of appendicular stump Figure A: The appendiceal stump with edematous and erythematous changes is visualized. Figure B: Resected appendiceal stump; the stump length intraoperatively was 0.67 cm (measured with AI).

Based on the clinical presentation of acute abdomen, imaging findings, and his prior history of appendectomy, an alternative etiology was initially suspected, prompting an open surgical approach. Regarding the surgical procedure performed: An infraumbilical incision was made, with dissection through the layers until entering the right abdominal cavity, where an edematous and erythematous appendiceal stump was found, confirming the diagnosis of stump appendicitis (Figure 2A). The stump was ligated at its base, the inflammatory phlegmon was dissected, and stump appendectomy was performed (Figure 2B) using the Halstead technique. The abdominal cavity was then irrigated, followed by layered closure, and the procedure was concluded. Diagnostic laparoscopy was not performed, as the necessary equipment was unavailable at our hospital. If stump appendicitis had been considered from the outset and laparoscopic access had been possible, a laparoscopic appendectomy would have been the preferred approach, offering faster recovery and lower morbidity and mortality. 

Outcome and follow-up

**Figure 3:**
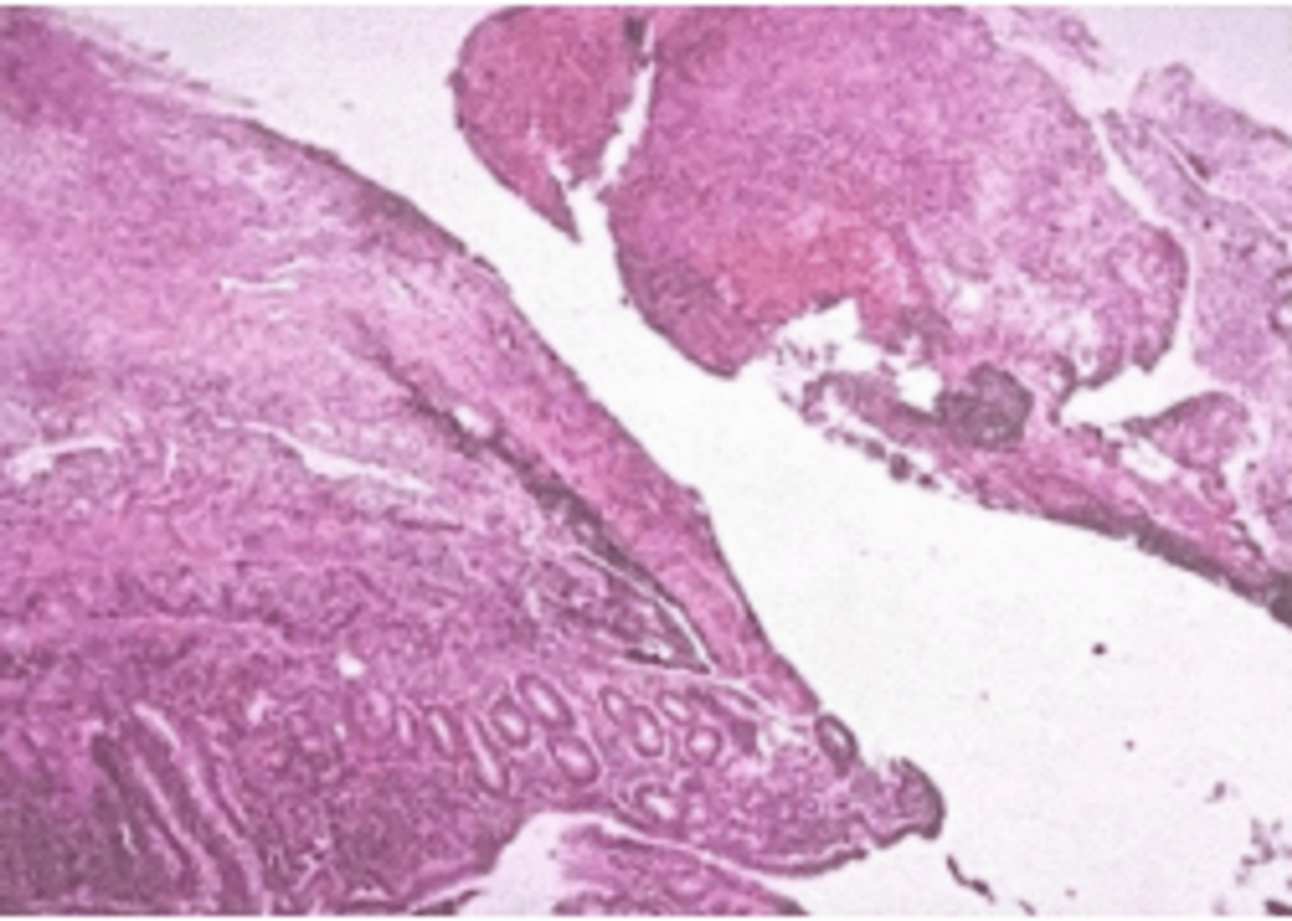
Histological section Histological sections corresponding to the cecal appendix showing acute inflammatory changes.

On the day following surgery, the patient showed good postoperative progress; therefore, discharge home was indicated with analgesic and antibiotic treatment. Weeks later, the histopathological diagnosis was obtained, revealing acute fibrinopurulent appendicitis with purulent peritonitis associated with a 4x4 cm phlegmon (Figure 3).

One month later, the patient was evaluated in a follow-up consultation, showing continued good clinical progress, and was discharged from surgical care.

## Discussion

Acute appendicitis is a common surgical diagnosis worldwide, with approximately 250,000 appendectomies performed annually in the United States [8]. A rare but notable complication is stump appendicitis, which occurs when the residual appendiceal stump is left too long during initial surgery. Although the exact etiology is unclear, its reported incidence is approximately 1 in 50,000 cases and is likely underreported [9].

Clinical presentation

Symptoms mimic classic appendicitis, most commonly right lower quadrant (RLQ) pain. Onset may occur from days to years’ post-appendectomy. Rare presentations include atypical abdominal pain, chronic draining sinuses, or distant abscesses. Notably, approximately 20% of patients report previous RLQ pain episodes that did not prompt medical attention [9].

Imaging

Ultrasound serves as an initial screening tool, but CT is preferred due to superior specificity. CT can assess stump length, detect anatomical variants, identify retained appendicoliths, and locate postoperative abscesses. In pediatric populations, careful consideration of radiation exposure is necessary [10].

Preventive measures during initial appendectomy

Ensuring complete resection of the appendix with careful identification of the appendiceal base is essential. Key preventive strategies include laparoscopic visualization of the cecal base and secure closure with endoloops or staplers (when the equipment is available) and attention to anatomical variants (e.g., retrocecal or subserosal appendix). Ideally, the stump length should be <5 mm to reduce recurrence risk [8-10] (Table 1).

**Table 1:** Preventive measures during initial appendectomy

Preventive Measure	Description/Details	Benefit
Clear identification of the cecum	Visualize the base of the appendix before resection	Prevents leaving a long or residual stump [8]
Complete appendix resection	Remove the entire visible portion of the appendix	Reduces risk of future inflammation [9]
Minimal stump length	Ideally <5 mm	Decreases incidence of stump appendicitis [10]
Use of endoloops or staplers	Secure closure of the stump	Prevents postoperative leaks or perforation [8]
Inspection for anatomical variations	Retrocecal, subserosal, or duplicated appendix	Identifies situations at risk for incomplete resection [9]
Abdominal lavage and final inspection	Ensure hemostasis and absence of residual tissue	Minimizes postoperative complications [8]

Management

Treatment typically involves simple resection of the appendiceal stump; in severe cases with adjacent visceral involvement, a right hemicolectomy may be required [8].

## Conclusions

During the evaluation of any patient presenting with right lower quadrant abdominal pain and signs of acute abdomen with a history of appendectomy, the possibility of developing stump appendicitis should not be overlooked. It must always be considered as a differential diagnosis, as it can delay early treatment and allow the condition to progress to a life-threatening stage. Non-contrast CT remains the gold standard for diagnosis, along with clinical presentation. It is recommended that all surgeons performing appendectomies aim to leave the appendiceal stump shorter than 0.5 cm to reduce the risk of developing stump appendicitis.
